# Modulation of bacterial outer membrane vesicle production by envelope structure and content

**DOI:** 10.1186/s12866-014-0324-1

**Published:** 2014-12-21

**Authors:** Carmen Schwechheimer, Adam Kulp, Meta J Kuehn

**Affiliations:** Department of Biochemistry, Duke University Medical Center, Durham, NC 27710 USA; Department of Molecular Genetics and Microbiology, Duke University Medical Center, Durham, NC 27710 USA

## Abstract

**Background:**

Vesiculation is a ubiquitous secretion process of Gram-negative bacteria, where outer membrane vesicles (OMVs) are small spherical particles on the order of 50 to 250 nm composed of outer membrane (OM) and lumenal periplasmic content. Vesicle functions have been elucidated in some detail, showing their importance in virulence factor secretion, bacterial survival, and biofilm formation in pathogenesis. Furthermore, OMVs serve as an envelope stress response, protecting the secreting bacteria from internal protein misfolding stress, as well as external envelope stressors. Despite their important functional roles very little is known about the regulation and mechanism of vesicle production. Based on the envelope architecture and prior characterization of the hypervesiculation phenotypes for mutants lacking the lipoprotein, Lpp, which is involved in the covalent OM-peptidoglycan (PG) crosslinks, it is expected that an inverse relationship exists between OMV production and PG-crosslinked Lpp.

**Results:**

In this study, we found that subtle modifications of PG remodeling and crosslinking modulate OMV production, inversely correlating with bound Lpp levels. However, this inverse relationship was not found in strains in which OMV production is driven by an increase in “periplasmic pressure” resulting from the accumulation of protein, PG fragments, or lipopolysaccharide. In addition, the characterization of an *nlpA* deletion in backgrounds lacking either Lpp- or OmpA-mediated envelope crosslinks demonstrated a novel role for NlpA in envelope architecture.

**Conclusions:**

From this work, we conclude that OMV production can be driven by distinct Lpp concentration-dependent and Lpp concentration-independent pathways.

**Electronic supplementary material:**

The online version of this article (doi:10.1186/s12866-014-0324-1) contains supplementary material, which is available to authorized users.

## Background

Outer membrane vesicles (OMVs) bud from the outer membrane (OM) of Gram-negative bacteria [1-4]. These spherical particles are composed of outer membrane entrapping lumenal periplasmic content [3] and have a diameter of around 50 to 250 nm, as visualized by electron and atomic force microscopy [4,5]. Predominately, studies of OMV function have centered around topics related to pathogenesis, such as their role in the dissemination of virulence factors and genetic material, as well as degradation enzymes (proteases, hydrolases and lipases) which allow protection of an ecological niche and acquisition of nutrients in addition to the nucleation of biofilms [2,6-9]. OMV production is also an envelope stress response and a reduction in vesiculation under stressful conditions is harmful to the bacterial cells [10-17]. Our understanding of the mechanism and regulation of OMV production, however, remains extremely fragmented.

The Gram-negative envelope consists of a cytoplasmic or inner membrane (IM) and the OM, separated by the periplasmic space which contains the peptidoglycan (PG) sacculus [18]. The OM of Gram-negative bacteria is asymmetric with the inner leaflet composed of phospholipids and the outer leaflet composed of lipopolysaccharide (LPS) [19-21]. The PG is a highly dynamic polymer, especially during cell growth and growth phase transitions [22]. For envelope stability, the OM is tethered to the PG sacculus via an abundant OM lipoprotein, Lpp, by covalent crosslinking [23-26].

It has been long-appreciated that the OM must dissociate from the underlying PG for an OMV bud to form [27,28]. Indeed, the complete loss of envelope stabilizing factors leads to extremely high OMV production, although this is accompanied by a loss of membrane integrity and cellular leakage [4,29,30]. Since wild-type (WT) bacteria in normal and in inducing conditions, along with numerous hypervesiculation mutants, produce OMVs without compromising envelope stability [12,15,17,31-33], a more moderate and regulated modulation of envelope structure must be present that can yield OMVs.

We hypothesized that alterations in the PG structure underlying the OM could be a means by which cells may modulate OMV production in either direction. This idea is strengthened by data demonstrating that the deletion of the amidase autolysin in *Porphyromonas gingivalis*, an enzyme that cleaves PG amide bonds, led to an increase in OMV production [34]. The opposite effect, however, that increased crosslinking leads to hypovesiculation, has never been observed.

The IM lipoprotein, NlpA is one of very few envelope components that have been characterized and found to have a dominant effect on OMV production. It was previously established that the loss of NlpA caused decreased OMV production in an otherwise WT strain [15,31], and suppressed the protein accumulation-driven hypervesiculation phenotype of the Δ*degP* mutant, which lacks the periplasmic protease/chaperone DegP [15].

In this study, we analyzed the effect on OMV production of mutations that alter PG structure and Lpp crosslinking. We were also curious whether bound Lpp levels dictate vesiculation levels for bacteria under inducing conditions, particularly those involving build-up of material in the periplasm. We investigated bound Lpp levels for mutants in which periplasmic misfolded protein, PG fragments, or LPS accumulation led to upregulated OMV production. Finally, we investigated the genetic interactions between *nlpA* and genes encoding envelope modifying and stabilizing proteins.

## Results

### OMV production and Lpp crosslinking changes inversely with altered PG structure

**Figure 1:**
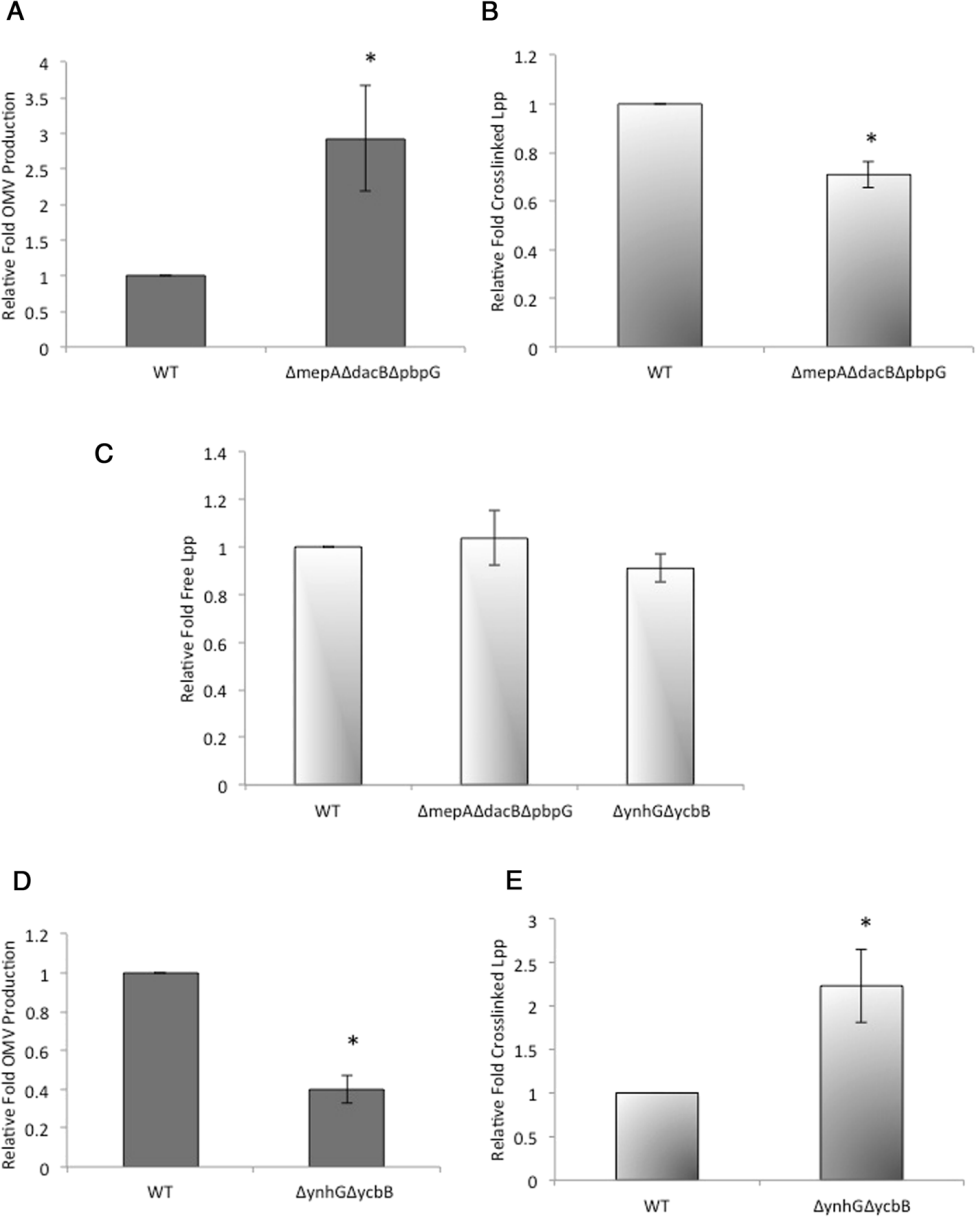
**OMV production and Lpp crosslinking changes inversely with altered PG structure. (A)** Relative fold OMV production in cultures of the indicated strains grown in LB overnight at 37°C was determined by quantitating OMVs by OMPs, normalizing to OD_600_, and dividing by OD_600_-normalized OMV production in a WT culture. **(B)** Relative fold crosslinked Lpp in cultures of the indicated strains grown in LB to an OD_600_ of ~ 0.4 at 37°C was determined by immunoblotting of PG copurified Lpp, normalizing to OD_600_, and dividing by OD_600_-normalized crosslinked Lpp in a WT culture. **(C)** Relative fold of free Lpp in cultures of the indicated strains grown overnight in LB was determined by quantitative immunoblotting of Lpp in whole cell preparations, normalizing to OD_600_, and dividing by OD_600_-normalized Lpp in a WT culture. **(D)** Relative fold OMV production in cultures of the indicated strains grown in LB overnight at 37°C was determined as in part A. **(E)** Relative fold crosslinked Lpp in cultures of the indicated strains grown in LB to an OD_600_ of ~ 0.4 at 37°C was determined as in part B. Error bars indicate standard error of the mean (SEM). p values refer to comparisons with WT. *, p ≤ 0.05; n ≥ 3.

To examine the relationship between modulation of PG structure and levels of OMV production, we examined a PG hydrolase mutant, Δ*mepA*Δ*dacB*Δ*pbpG*, which lacks three of the endopeptidases that cleave the PG peptide bonds. We observed that OMV production increased in this triple mutant strain (Figure 1A). This strain, along with all other strains used in this work, were tested for membrane integrity using previously published assays [15] (Additional file 1: Table S1) so that we could be sure that the measured OMV fractions were not inflated with the presence of membrane fragments released as a consequence of instability.

Next, we examined the Lpp crosslinking levels of the Δ*mepA*Δ*dacB*Δ*pbpG* strain. We used an immunoblotting assay that allows us to distinguish between the PG crosslinked form of Lpp, and the OM lipid-anchored but uncrosslinked form of Lpp (historically referred to as the ‘bound’ and the ‘free’ form, respectively). As expected, we found an inverse relationship between OMV production and bound Lpp (Figure 1B). The amount of free Lpp was comparable to WT (Figure 1C), suggesting that the observed decrease is not a result of an overall decrease in Lpp.

We also investigated the L,D-transpeptidase Δ*ynhG*Δ*ycbB* double mutant, which contains the common D-Alanine (D-Ala)-Diaminopimelic acid (DAP) peptide crosslinks but lacks the minor DAP-DAP crosslinks [35]. We were especially interested in this strain in light of its relationship to Lpp crosslinking, since it had been shown that DAP-DAP muropeptides are enriched in covalently crosslinked Lpp [36]. Interestingly, the loss of DAP-DAP crosslinks correlated with a strong hypovesiculation phenotype, ~ 60% lower than WT (Figure 1D). When examining the Lpp crosslinking levels of this mutant we found a significant increase in covalently attached Lpp (by ~ 2.6 fold, Figure 1E), whereas the concentration of free Lpp resembled WT levels (Figure 1C). It should be noted that this was the first strain in which we observed an increase in bound Lpp, demonstrating that bound Lpp levels can have a dynamic range in both directions. Taken together, these data suggest that the modulation of PG structure can alter the levels of OMV production in either direction via an inverse relationship to PG-Lpp crosslinking.

### The inverse relationship between Lpp crosslinks and OMV production does not hold for mutants that accumulate periplasmic protein

**Figure 2:**
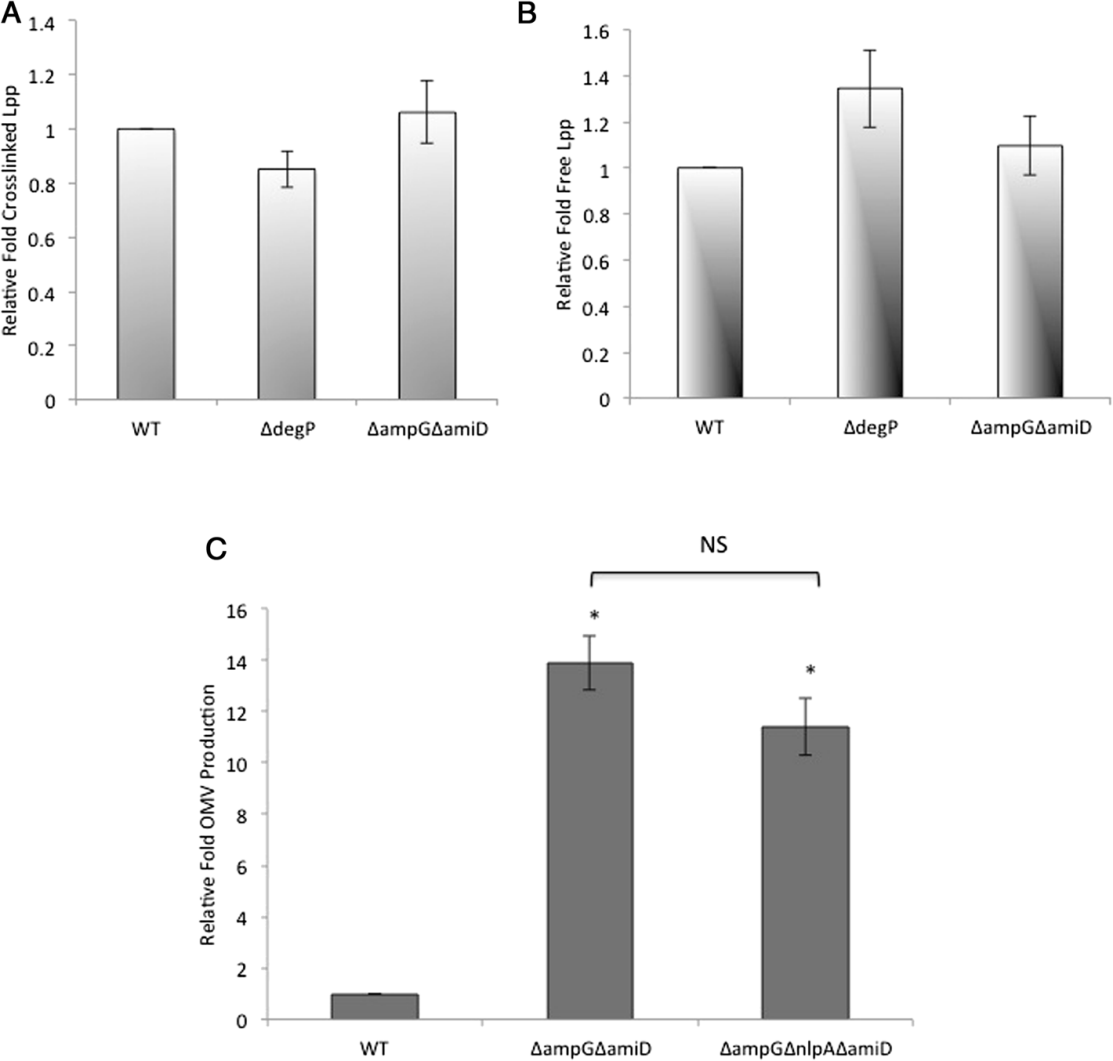
**Accumulation periplasmic PG fragments or protein correlates with increased vesiculation without alteration of Lpp crosslinking. (A)** Relative fold crosslinked Lpp in cultures of the indicated strains grown in LB to an OD_600_ of ~ 0.4 at 37°C was determined as described in Figure 1B. **(B)** Relative fold of free Lpp in cultures of the indicated strains grown overnight in LB at 37°C was determined as described in Figure 1C. **(C)** Relative fold OMV production in cultures of the indicated strains grown in LB overnight at 37°C was determined as described in Figure 1A. Error bars indicate SEM. p values refer to comparisons with WT unless indicated by a bracket. *, p ≤ 0.05; NS, p > 0.05; n ≥ 3.

The data presented above demonstrated that for strains containing mutations directly affecting PG structure, vesiculation inversely correlates with the cellular concentration of Lpp crosslinks. We were curious whether we would observe a decrease in the level of covalent Lpp crosslinking when increased OMV production was induced by misfolded protein build-up, as is the case in the ~40-fold OMV hypervesiculating Δ*degP* mutant [15]. We quantified covalent Lpp crosslinks in the Δ*degP* strain and found that Lpp crosslinking levels were not different from the WT (Figure 2A). In control experiments, free Lpp in the mutant were also not statistically significant from WT levels (Figure 2B). These data suggest that the accumulation of periplasmic protein creates an increase in periplasmic pressure, which in turn leads to hypervesiculation, but that this occurs without altering the total numbers of Lpp crosslinks.

### Accumulation of PG fragments correlates with increased OMV production without altering Lpp crosslinking

We further examined vesiculation and envelope crosslinking in another case where envelope products accumulate in the periplasm. The Δ*ampG* mutant lacks the IM permease AmpG, and this mutant is impaired in transporting muropeptides from the periplasm to the cytoplasm for PG recycling [37]. We utilized the Δ*ampG*Δ*amiD* double mutant which also lacks the amidase AmiD, causing large PG fragments to accumulate in the periplasm because they are also too large to fit through the porins [38]. When we examined OMV production, we determined that the Δ*ampG*Δ*amiD* mutant exhibited ~ 14-fold increased OMV production with respect to WT (Figure 2C). These data supported the hypothesis that periplasmic accumulation of PG fragments caused their subsequent shedding into the medium via OMVs. Direct verification and quantitation of PG fragments in the OMVs is extremely challenging, technically, and therefore was not able to be determined in the scope of this study. To investigate the state of the envelope for this strain, free and crosslinked Lpp levels were measured. No difference in the levels of bound or free Lpp were found for the Δ*ampG*Δ*amiD* mutant and the WT strain (Figure 2A and B). In sum, a mutant in which PG fragments accumulate in the periplasm hypervesiculates without exhibiting altered total levels of Lpp crosslinking, similar to the effect when protein accumulation drives vesiculation.

### LPS accumulation also leads to hypervesiculation without modulating bound Lpp concentration

We next reasoned that accumulation of LPS fragments could generate a similar effect to induce OMV production as either the accumulation of PG fragments or periplasmic protein. Data published recently indicate that individual mutations that alter the sugar core structure of LPS (Δ*rfaC,* Δ*rfaG,* and Δ*rfaP*) lead to periplasmic LPS accumulation due to the disruption of LPS maturation in the envelope of the cell [39]. Additional evidence further supports the concept of periplasmic LPS accumulation: *rfaC* and *rfaG* mutant strains contain an increased amount of LPS in comparison to WT [40,41], and furthermore increasing LPS production leads to abnormal structures in the periplasm, implying that LPS overproduction results in a reduction of proper, OM-localized LPS, but not a reduction in the overall amount of LPS in the envelope [42].

**Figure 3:**
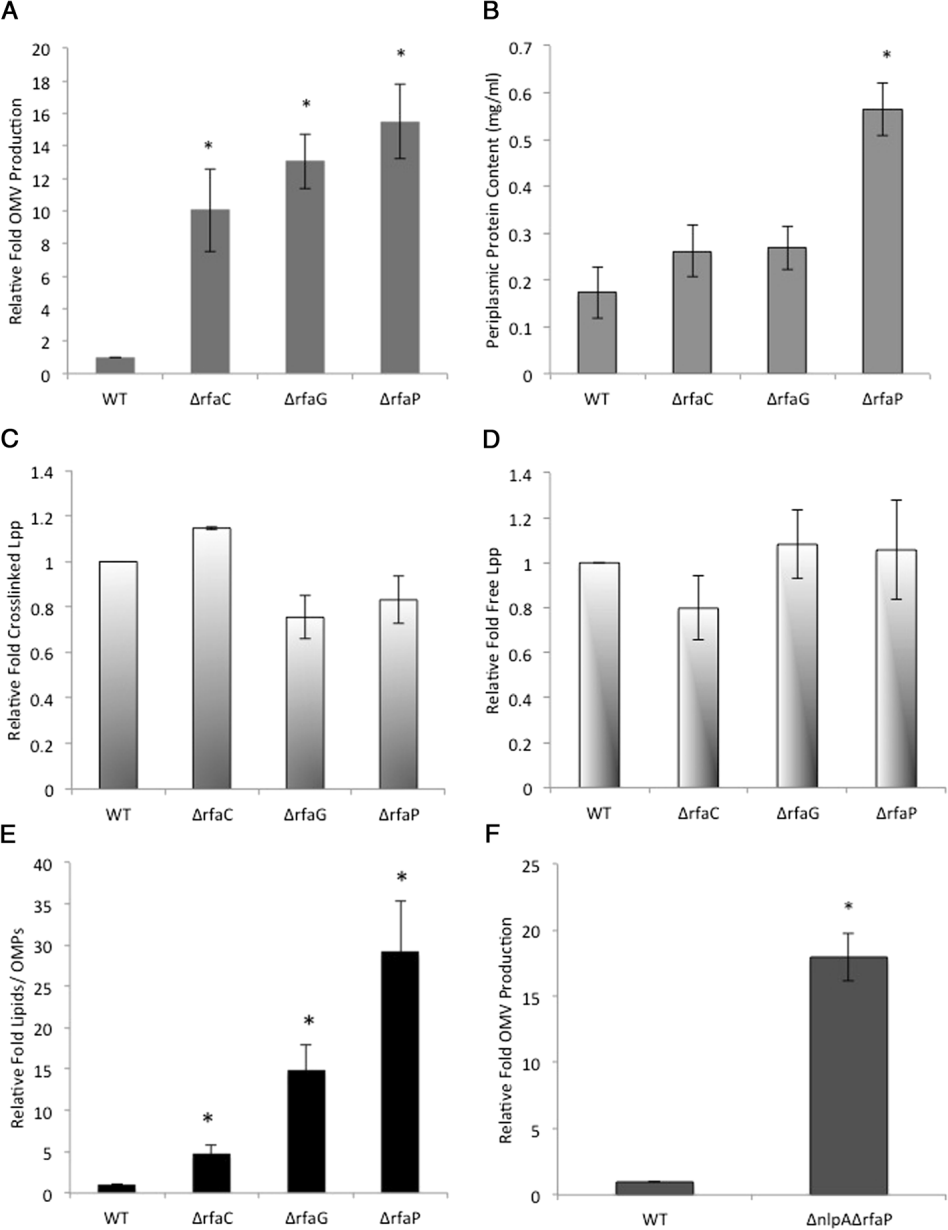
**Accumulation of periplasmic LPS correlates with increased OMV production without alteration of Lpp crosslinking. (A)** Relative fold OMV production in cultures of the indicated strains grown in LB overnight at 37°C was determined using FM4-64 and normalized to CFU as in Figure 1A. **(B)** Protein concentrations in periplasm preparations of the indicated strains grown ~16-18 hrs in LB at 37°C were determined by Bradford Assay. **(C)** Relative fold crosslinked Lpp in cultures of the indicated strains grown in LB to an OD_600_ of ~ 0.4 at 37°C was determined as described in Figure 1B. **(D)** Relative fold of free Lpp in cultures of the indicated strains grown in LB at 37°C was determined as described in Figure 1C. **(E)** The lipid to protein ratio in the OMVs purified from cultures of the indicated strains grown in LB overnight at 37°C was determined by dividing the amount of lipid, measured using FM4-64, by the OMP concentration, measured by densitometry. **(F)** Relative fold OMV production in cultures of the indicated strains grown in LB overnight at 37°C was determined as described in Figure 1A. Error bars indicate SEM. *, p ≤ 0.05; n ≥ 3.

As expected, all three LPS core mutants exhibited hypervesiculation phenotypes (Figure 3A). However, it is recognized that mutant strains Δ*rfaC,* Δ*rfaG,* and Δ*rfaP* activate the σ^E^ envelope heat shock response, a process discovered to require both mislocalized, periplasmic LPS as well as a misfolded outer membrane protein (OMP) component for activation [39]. Since σ^E^ activation implicated the presence of misfolded OMPs, and previous work from our lab showed that periplasmic protein accumulation leads to hypervesiculation, we needed to determine if the reason for hypervesiculation in the Δ*rfaC,* Δ*rfaG,* and Δ*rfaP* mutants was not actually solely due to increased periplasmic protein levels. We measured the amount of periplasmic protein in the mutants and found that the Δ*rfaC* and Δ*rfaG* mutants contained WT levels (Figure 3B), supporting the hypothesis that it was the increase in periplasmic LPS, not protein, which led to hypervesiculation. The periplasmic protein concentration in the Δ*rfaP* strain was significantly higher than that of the WT (Figure 3B), thus we could not distinguish whether the hypervesiculation phenotype of this mutant resulted from accumulation of periplasmic protein or LPS, or a combination of these.

Next, we determined the amount of covalently crosslinked Lpp in these LPS mutants in order to see if these inversely correlated with the OMV phenotypes. Covalent Lpp crosslinking was unchanged with respect to WT for Δ*rfaC*; Δ*rfa*G and Δ*rfaP* exhibited a slight reduction, albeit not statistically significant (Figure 3C). In control experiments, the amount of free Lpp in the strains was also not significantly different from WT (Figure 3D). These results support the hypothesis that OMV production in these LPS mutants is predominantly driven by accumulated material rather than a decrease in overall covalent Lpp crosslinking.

To test our model that the accumulation of mislocalized envelope lipid in the cell leads to its secretion via OMVs, we assessed whether OMVs produced by these LPS mutants were enriched in lipid. We quantified the lipids in OMVs using a lipophilic dye (FM4-64) that becomes fluorescent upon membrane intercalation. These values were then divided by the quantity of OMPs in the OMVs from each of the strains. The results show a four-fold increase in the lipid to OMP ratio for Δ*rfaC* OMVs*,* a 15-fold increase for Δ*rfaG,* and a 29-fold increase for Δ*rfaP* OMVs, with respect to the WT OMV control, confirming lipid accumulation in the OMVs of Δ*rfaC*, Δ*rfaG,* and Δ*rfaP* (Figure 3E). These data strongly support the idea that accumulated lipid, LPS, is in the secreted OMVs.

### A complex role for NlpA in envelope architecture

To further investigate the envelope architecture of periplasmic accumulation-induced hypervesiculating mutants, we tested the effect of deleting *nlpA* in those strains. The loss of the IM lipoprotein, NlpA*,* decreases OMV production in an otherwise WT strain [15,31] and suppresses the hypervesiculation phenotypes of the protein-accumulating, Δ*degP* mutant [15]. Interestingly, however, the Δ*nlpA* mutation was not epistatic to Δ*ampG*Δ*amiD,* and the Δ*nlpA*Δ*rfaP* double mutant still produced a significantly increased amount of OMVs (Figures 2C and 3F). These data suggested that membrane architecture differs for hypervesiculating strains containing different accumulated periplasmic products.

**Figure 4:**
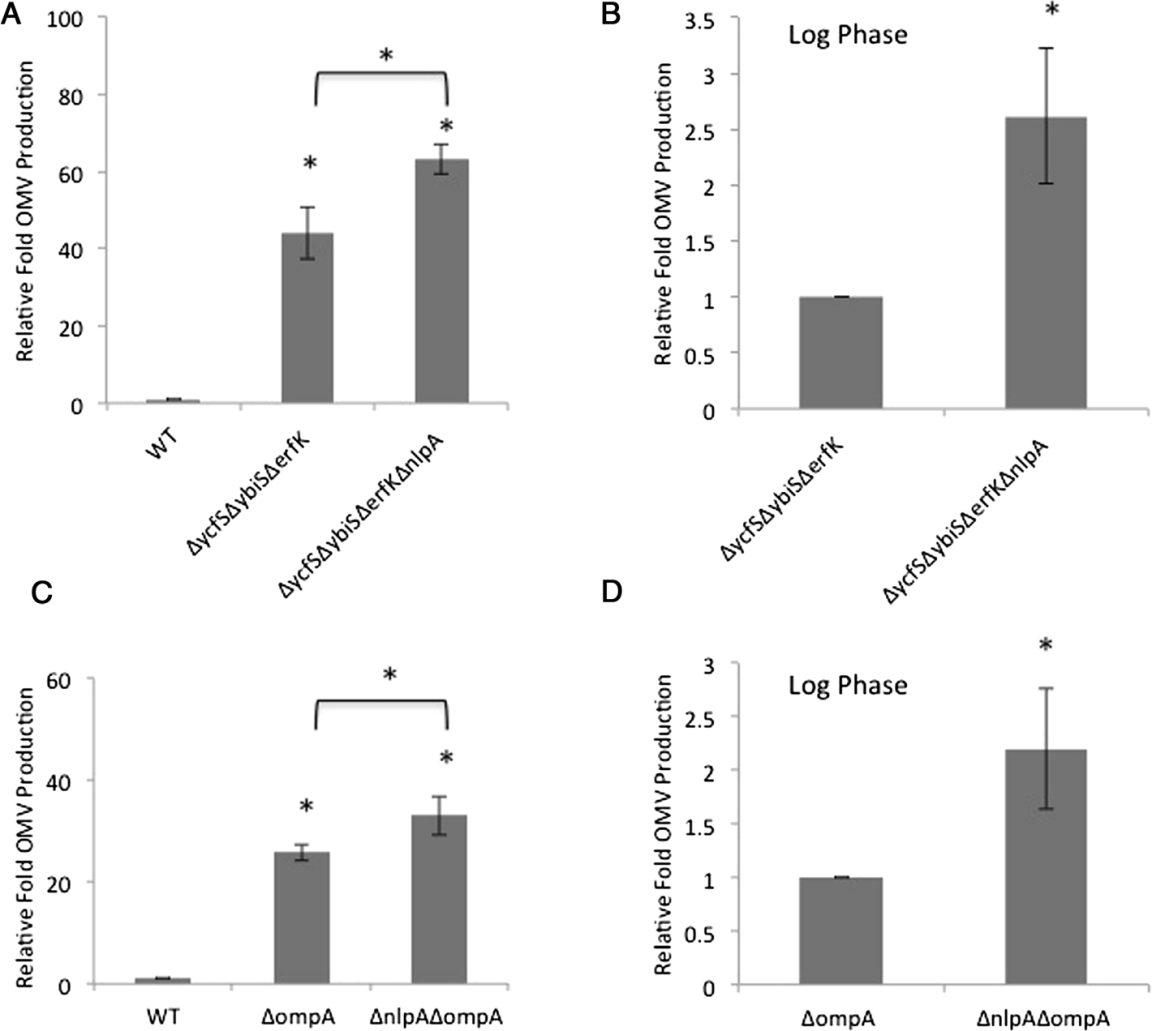
**The effect of Δ** ***nlpA*** **on vesiculation phenotypes.** Relative fold OMV production in cultures of the indicated strains grown in LB at 37°C overnight **(A, C)** or to an OD_600_ of ~ 0.4 **(B, D)** was determined as described in Figure 1A. p values refer to comparisons with WT **(A, C)** or indicated background strain **(B, D)**. Error bars indicate SEM. Statistical comparisons are with WT **(A, C)** or mutant control strains **(B, D)** unless denoted by a bracket. *, p ≤ 0.05; n ≥ 4.

We next examined the relationship between *nlpA* and *lpp*. We initially constructed and characterized a Δ*nlpA*Δ*lpp* double mutant. We found that Δ*nlpA* appeared to have no effect on the phenotype of the Δ*lpp* strain (Additional file 1: Figure S1), however the high level of “vesicles” produced by either the *lpp* mutant or the double mutant are probably not true OMVs. The loss of Lpp, the most abundant *E. coli* protein [18,24], makes the envelope of this strain quite fragile [23,29,30], and it is likely that any additional structural stress (e.g. from the *nlpA* deletion) would be negligible. Therefore, we turned to the triple L,D-transpeptidase mutant Δ*ycfS*Δ*ybiS*Δ*erfK* which lacks the three enzymes which from the covalent crosslink between Lpp and PG [43] and exhibits a hypervesiculation phenotype (43-fold) [4] (Figure 4A). The addition of the *nlpA* mutation to the triple mutant led to an increase in vesiculation (Figure 4A). Since we previously demonstrated that the loss of *nlpA* does not manifest its OMV phenotype until stationary phase [15], we were curious to examine whether OMV production in the triple mutant was unaffected by the lack of *nlpA* during log phase growth. To test this, we quantified OMVs in the supernatant of log-phase cells and found that the deletion of *nlpA* increased vesiculation in the Δ*ycfS*Δ*ybiS*Δ*erfK* L,D-transpeptidase mutant (Figure 4B). These results supported the concept that *nlpA* plays a critical role in stabilizing the envelope under particular conditions.

We also examined whether the loss of *nlpA* showed genetic interactions with other envelope stabilizing factors. Previous genetic and crosslinking studies have shown that there is an interaction between Lpp and OmpA [30,44,45], an OM β-barrel protein with a periplasmic PG-interaction domain [46,47]. A deletion in *ompA*, which encodes OmpA, resulted in ~26-fold hypervesiculation (Figure 4C), consistent with the phenotypes of the Δ*ompA Salmonella* and *Vibrio cholerae* mutants [29,48]. We tested if the *nlpA* deletion was epistatic to Δ*ompA.* Similar to the results for the triple L,D-transpeptidase mutant, the Δ*nlpA* mutation also exacerbated the Δ*ompA* hypervesiculation phenotypes in both overnight (Figure 4C) and log phase cultures (Figure 4D). Together, these data support a complex role for NlpA, depending on envelope conditions. Specifically, NlpA is critical to the ability to increase OMVs in conditions of protein, but not PG fragment and lipid accumulation, and the loss of NlpA increases hypervesiculation when levels of envelope stabilizing factors are decreased.

## Discussion

**Figure 5:**
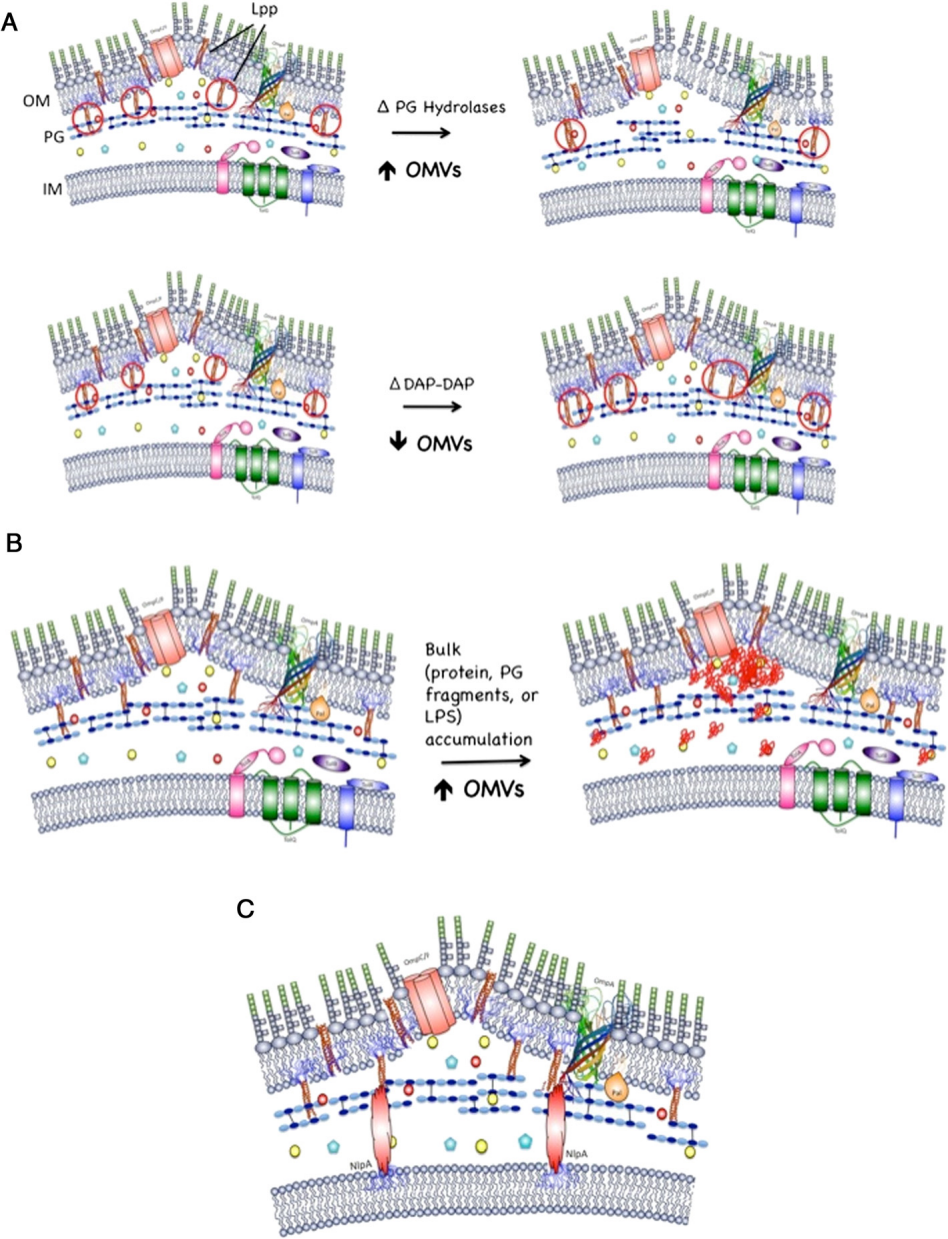
**Mechanistic working models of OMV production and modulation.** Models of how changes in envelope structure lead to modulation of OMV production are based on the data presented here and in prior studies, as described in the text. **(A)** Modulation of PG structure and metabolism can up- and downregulate OMV production through levels of bound Lpp (circled in red). **(B)** Periplasmic bulk accumulation (red aggregates) leads to hypervesiculation without altering bound Lpp levels. **(C)** The IM anchored lipoprotein NlpA contributes to envelope integrity in conjunction with Lpp and OmpA.

Despite investigations revealing that OMVs function in critical areas such as pathogenesis, bacterial survival, and envelope stress, our knowledge of the mechanism and regulation of OMV production has remained quite cryptic. To gain mechanistic insight into OMV production, we analyzed the effect of specific gene mutations on OMV phenotypes and their relationship to cell envelope structure. The results begin to reveal a complex relationship between envelope remodeling, crosslinking, periplasmic content, and OMV production. We have shown here that multiple routes modulate vesiculation: one that is dependent on and one that is independent of the overall concentration of bound Lpp. Both of these pathways appear to be stimulated by multiple factors: cellular covalent Lpp crosslinking can be altered by changes in PG structure, whereas envelope accumulation of material (protein, PG fragments and LPS, or a combination of these), as well as the loss of the IM lipoprotein NlpA in a background lacking bound Lpp or OmpA, result in hypervesiculation with minimal or no contribution from overall changes in bound Lpp levels. The data are summarized in a set of working models in Figure 5.

### Fine-tuning of OMV production through PG biosynthesis and structure

The level of Lpp crosslinking was investigated for mutants with moderate PG structure effects that also exhibited increased and decreased levels of vesiculation. Here we report that the triple endopeptidase deletion mutant hypervesiculates and exhibits a decrease in covalent Lpp crosslinking (Figure 1B). This hypervesiculation phenotype was notably consistent with the previous *P. gingivalis* endopeptidase mutant [34]. The opposite situation was found with the loss of the genes responsible for the minor DAP-DAP PG crosslinks (Δ*ynhG*Δ*ycbB*). In this mutant, OMV production is lower than WT (Figure 1D), with a concomitant increase in bound Lpp (Figure 1E). These data support a model in which PG dynamics directly modulate the number of covalent envelope crosslinks and, thereby, indirectly modulate OMV production (Figure 5A).

The increase in bound Lpp for the Δ*ynhG*Δ*ycbB* strain was particularly interesting in light of a previous report which showed that PG-Lpp crosslinks are enriched at sites of DAP-DAP crosslinks [36]. We hypothesize that either DAP-DAP crosslinks could serve as “location markers” for crosslinking of Lpp and that in the absence of these markers, Lpp crosslinking to the PG is more random and more distributed across the PG sacculus, or alternatively, that the residues typically involved in the DAP-DAP crosslinks may be utilized for Lpp crosslinking in this mutant.

### OMV production relieves stress caused by the accumulation of diverse, potentially harmful products in the envelope

Here we demonstrate that the accumulation of periplasmic PG fragments and LPS leads to an increase in OMV production. These data are consistent with the previously described role of OMVs in relieving protein-mediated envelope stress induced by a σ^E^-stimulating model misfolded polypeptide and the lack of the DegP protease [12,15]. In addition, we detected increased ratios of lipid:protein in LPS mutant strain OMVs, which indicates accumulated LPS cargo enrichment in OMVs. Similarly, the σ^E^-stimulating model misfolded polypeptide was enriched in OMVs, and misfolded DegP substrates were present in OMVs purified from the DegP protease-deficient strain [12,15]. PG in OMVs from PG accumulating strains could not be detected due to technical limitations, however it should be mentioned that the Δ*ampG*Δ*amiD* mutant strain releases large PG fragments into the cell-free medium [38], and since these are too large to diffuse through the OM porins, this observation is consistent with their secretion via OMVs.

We have previously found that vesiculation enhances survival in cases of periplasmic protein accumulation [12,15] and can now extend this model to include the shedding of LPS via OMVs. Very recently, YciM was identified as a negative regulator of LPS biosynthesis, and an excess of LPS was confirmed to be responsible for the death of *yciM* mutants [49]. Interestingly, they report that suppressor mutations include those that either downregulate LPS biosynthesis via other routes, or they are part of a group of genes that is involved in OM assembly or organization (*lpp*, *rfaP*, *ybcN*, *galU*). Notably, all the mutants from the second group hypervesiculate (A. Kulp, A. Manning, B. Sun, T. Ai, D. Rodriguez, A. Schmidt, and M. Kuehn, unpublished data) [29,50].

These results further establish the general and important role OMV production plays in bacterial well-being, but we considered why bacteria do not simply expand their periplasm to accomodate the excess material without the concomitant loss of “macromolecular energy” that results from OMV release. In fact, it has been shown that the eukaryotic endoplasmic reticulum membrane expands to adapt to an increase in misfolded protein [51]. The answer is straightforward when considering the bacterial envelope architecture: The OM and PG are connected by Lpp, a finite covalent crosslink. With such a constraint, either the concentration of misfolded/mislocalized envelope material could increase, the level of crosslinks could decrease, or the membrane could bulge out. High concentrations of material could become toxic to the proper function of the envelope cells [15,52], therefore this is not a viable option. Unlike the situation for PG structural mutants, overall bound Lpp levels do not change under conditions of periplasmic accumulation, suggesting more localized changes in the envelope architecture were responsible for OMV generation (see model, Figure 5B). Apparently, the trapped periplasmic material cannot prevent the formation of bound Lpp, but instead pushes the OM outward, either by taking advantage of “nanoterritories” of OM containing locally decreased levels of bound Lpp, or by displacing bound Lpp to sites on the periphery of the outwardly bulging OM. Subsequent spontaneous membrane fusion events, could then result in OMV budding and release.

### The contribution of NlpA to envelope architecture

The data demonstrating that the loss of *nlpA* increased OMV production in strains that were also missing the envelope stabilizing factors, bound Lpp and OmpA, (Figure 4) led us to hypothesize a structural role of the IM lipid-anchored protein, NlpA, within the envelope that depended on these other factors: NlpA could provide an IM-based scaffolding site to stabilize the sites of Lpp- and OmpA-based envelope crosslinks as depicted in our working model (Figure 5C). This is supported by the observations that NlpA is most critical during stationary phase [15], at a time when Lpp-PG crosslinking has been shown to increase [36]. But, if NlpA helps to stabilize crosslinks, why would the Δ*nlpA* strain then have a hypovesiculation phenotype? We propose that other factors in the envelope that depend on bound Lpp or OmpA are overcompensating for the loss of *nlpA* in this mutant, creating a more tightly crosslinked envelope. Interestingly, the undervesiculation phenotype of the Δ*nlpA* strain is manifested in stationary phase, whereas the phenotypes presented in this work are already present in log phase, suggesting that the factor in the Δ*nlpA* strain that can (over)compensate for NlpA only appears late in the cell cycle. Notably, vesiculation levels did not change when *nlpA* was deleted in mutants that directly affect PG components (Δ*ampG*Δ*amiD and* Δ*nlpI*) (Figure 2C and Schwechheimer et al, [58]). Further work is necessary to fully elucidate the accessory role of NlpA in the envelope and in OMV biogenesis.

## Conclusions

### Implications for regulated OMV production by WT bacteria

In sum, these data reveal that OMV levels are not solely dictated by Lpp crosslinking; at least two mechanisms can alter OMV budding, one dependent on and the other independent of overall levels of Lpp crosslinking. Our results help us to understand how WT bacteria might regulate OMV levels in different situations and times in their life cycle. In the first, cells could use localized or cell-cycle (temporal) modulation of the PG structure by modifying the equilibrium between PG synthesis and degradation to affect overall bound Lpp and, consequently, OMV levels. In the other, bulk deposition of envelope material within the periplasm, as a result of a localized secretion apparatus or a stress response, could allow outward bulging of the OM and ultimately OMV release at areas with locally-reduced amounts of bound Lpp or by relocating bound Lpp. As a complex entity whose integrity must be preserved for the viability of the cell, the envelope is modulated by numerous other factors, such as OmpA and NlpA, which contribute in specific ways to the modulation of the envelope architecture. Although many of the envelope components studied here are conserved amongst other Gram-negative bacterial species, further investigation is required to understand whether these principles regarding the modulation of OMV production are also conserved in other species.

## Methods

### Growth conditions and reagents

Strains used in this work are summarized in Table 1. Bacteria were grown in liquid culture in Luria–Bertani (LB) broth (EM Science) or on plates of solid LB agar supplemented with 50 mg/mL kanamycin or 100 mg/ mL ampicillin (Sigma). The single gene mutants originate from the Keio Collection [53]. To create mutants with multiple deletions, the kanamycin resistance marker was removed from the single mutant [54]. The additional mutation was then added by transduction of the marked gene deletion using P1 phage [55] from the donor single Keio mutant strain into the unmarked Keio recipient mutant strain. The single Keio deletion strains, as well as the mutants constructed for this work were either sequenced with a primer upstream and downstream of the deleted gene or PCR amplified with primers upstream/ downstream of the deleted gene and the kanamycin cassette to confirm the genotypes.

**Table 1:** **Strains used in this study**

**Strains**	**Genotype**	**Source/reference**
BW25113	*rrnB3 ΔlacZ4787 hsdR514 Δ(araBAD)567Δ(rhaBAD)568 rph-1*	WT of Keio collection (Baba et al. 2006 [53])
Keio collection single mutants	BW25113 with indicated single mutations: Δ*nlpA*::*Kan*, Δ*degP*::*Kan*, Δ*rfaC*::*Kan*, Δ*rfaG*::*Kan,* Δ*rfaP*::*Kan*	(Baba et al. 2006 [53])
MK1277	BW25113 Δ*ycfS*, Δ*ybiS*, Δ*erfK*::*Kan*	(Schwechheimer et al. 2013 [4])
MK1334	BW25113 Δ*ampG*, Δ*amiD*::*Kan*	This Work
MK1335	BW25113 Δ*ampG*, Δ*nlpA*, Δ*amiD*::*Kan*	This Work
MK1336	BW25113 Δ*pbpG*, Δ*dacB*, Δ*mepA*::*Kan*	This Work
MK1337	BW25113 Δ*ynhG*, Δ*ycbB*::*Kan*	This Work
MK1352	BW25113 Δ*ycfS*, Δ*ybiS*, Δ*erfK,* Δ*nlpA*::*Kan*	This Work
MK1353	BW25113 Δ*nlpA,* Δ*ompA::Kan*	This Work

### OMV purification and quantitation

Media (250 mL) was inoculated (1:250 dilution) from 37°C overnight cultures, and the bacterial cultures grown to an OD_600_ ~ 0.4 (for log phase) or grown overnight at 37°C (~16 h). Cells were pelleted with the Beckman Avanti J-25 centrifuge (JLA-10.500 rotor, 10 000 *g*, 10 min, 4°C) and the resulting supernatants filtered [low protein binding Durapore membrane, 0.45 μm polyvinylidene fluoride, Millipore]. Filtrates were centrifuged again with the Beckman Avanti J-25 centrifuge (JLA-16.250 rotor, 38 400 *g*, 3 h, 4°C) followed by another step of centrifugation with the Beckman Optima TLX Ultracentrifuge if the pellets were not visible. In these cases, most of the supernatant was poured off, and the region where pelleted material should be was “resuspended” in the residual supernatant and re-pelleted (TLA 100.3 rotor, 41 000 *g,* 1 h, 4°C). Pellets were resuspended in Dulbecco’s phosphate buffered saline with added salt (0.2 M NaCl) (DPBSS), and filter-sterilized through 0.45 μm Ultra-free spin filters (Millipore). A portion of the filtrate was plated on LB agar and incubated at 37°C overnight to verify that the suspensions were free of bacteria.

To quantitate OMV yield, OMV preparations were boiled for 6 min in 2× Laemelli buffer, separated by 15% SDS-PAGE, and stained with SYPRO Ruby Red (Molecular Probes) overnight in the dark. Prior to and after staining, the gel was fixed for 1 h in a solution of 10% MeOH and 7% acetic acid. Ruby-stained proteins were detected under UV light (Additional file 1: Figure S2 shows representative gels samples). *E. coli* Omps F/C and A were quantified by densitometry (NIH Image J software). The OMP density values were divided by the OD_600_ of the original culture to calculate OMV production and this value was divided by the OMV production of the WT or untreated control strain to determine relative fold OMV production. Measurements of OMV yield using FM4-64 was as described previously [31].

### PG purification, digestion and quantitation of covalently crosslinked Lpp

Unless otherwise indicated, media (500 mL) was inoculated (1:250 dilution) from overnight 37°C bacterial cultures and cultures grown at 37°C until they reached OD_600_ ~ 0.4. PG was isolated from broth cultures based on the protocol by Lam et al. [56]. Briefly, cells were pelleted and resuspended in PBS after which the ice-cold suspensions were dropped in an equal volume of vigorously stirring, boiling 10% SDS. Samples were boiled for 4 h and then incubated at 37°C, continuously shaking, overnight. The following day, the PG was pelleted with the Beckman Optima TLX Ultracentrifuge (TLA 100.3 rotor, 80 000 *g,* 15 min, 30°C), resuspended in 1% SDS followed by another 2 h of boiling. PG was washed four times with deionized water and finally resuspended in equal volumes of deionized water.

Equal fractions of the purified sacculi were digested with 15 mg/mL chicken egg lysozyme (Sigma-Aldrich) in 10 mM Tris–HCl, pH 8, at room temperature for 2 days. Lysozyme digested PG was separated by 15% SDS-PAGE and Lpp was detected by immunoblotting and quantified by densitometry (NIH Image J software). The Lpp density values were divided by the OD_600_ of the original culture to calculate the amount of Lpp that was covalently crosslinked to PG, and this value was divided by the PG-crosslinked Lpp of the WT strain to determine relative fold of bound Lpp. We chose to use cell density as the denominator for these experiments rather than the traditional total PG, since this calculation rather provides insights into the budding dynamics of the OM.

### Quantitation of free Lpp

This method was adapted from Cowles et al. [57]. A 5 ml culture was grown overnight (~16 hrs) in LB at 37°C. 1 ml of this culture was spun down in a microfuge (10 000 *g*, 4 min, room temperature), resuspended in 50 μl 1% SDS in PBS and 50 μl 2× Laemelli buffer. Samples were boiled for 10 min and separated by 15% SDS-PAGE. Free Lpp was detected by immunoblotting and quantified by densitometry (NIH Image J software). The free Lpp density values were divided by the OD_600_ of the original culture to calculate the amount of free Lpp, and this value was divided by the free Lpp of the WT control strain to determine relative fold of free Lpp.

### Periplasmic protein content

Periplasm was isolated and quantified after overnight growth (37°C, 16–18 h) using a previously published protocol [15].

### FM4-64 lipid analysis of OMVs

To determine the lipid to OMPs ratio within OMVs, one portion of the purified WT, Δ*rfaC*, Δ*rfaG*, and Δ*rfaP* OMVs were incubated with FM4-64 (Invitrogen), 3.3 g/ml in phosphate-buffered saline (PBS) for 10 min at 37°C. FM4-64 incubated in PBS was used as a negative control. The fluorescence signal was measured with a Molecular Devices SpectraMAX GeminiXS fluorometer (excitation: 506 nm, emission: 750 nm). To determine the OMPs concentration, a second portion of OMVs was treated as explained above under OMV purification and quantitation. Lastly the lipid value was divided by the OMP value and normalized to the WT strain.

### Statistics

Parameters used for the *T*-test are equal variance due to the comparison of identical experimental repetitions or unequal variance due to different experimental repetitions and a two-tail distribution. For direct sample size comparison, the paired *T*-test was used, and for fold comparison, the unpaired. The *T*-test value of ≤ 0.05 was considered statistically significant; if the value was lower than 0.05, the significance value is given under the corresponding data. The number of times each experiment was repeated (n) is stated in the figure legends.

## Additional file

Additional file 1:
**Supporting information.**
