# Supplementary material for: Modulation of bacterial outer membrane vesicle production by envelope structure and content
Source: BMC Microbiol. 2014 Dec 21;14:324. doi: 10.1186/s12866-014-0324-1 (PMC4302634; doi:10.1186/s12866-014-0324-1)
Supplement: Additional file 1: — Supporting information. [file 12866_2014_324_MOESM1_ESM.pdf]

Supporting Information

Experimental Procedures

To assess the effect of mutations on cell viability, cultures (5 mL) were inoculated to an OD_{600} ~0.03, cultures were incubated at 37°C for 6 h, and OD_{600} was measured hourly to assess growth for growth curves. OD_{600} at 6 h for WT cultures were compared with those of mutant cultures, respectively. Better, similar, or worse growth was noted if we found a difference $\geq$ 0.1 OD_{600} for at least 3 time points (Supplemental Table 1).

The sensitivity of cells to Actinomycin D was also used to assess membrane integrity defects [1, 2]. Cultures (25 mL) were grown overnight (37°C, 16-18 h) for stationary-phase, or to an OD_{600} for log phase cells. Actinomycin D (Sigma) dissolved in ethanol was added to 25 mL cultures to a final concentration of 5 μg/mL, cultures were incubated at 37°C, and OD_{600} was measured. Cultures were kept in the dark since Actinomycin D is light sensitive. OD_{600} at 6 h for WT (6h) were compared with those of mutant cultures. Better, similar, or worse growth was noted if we found a difference $\geq$ 0.1 OD_{600} for at least 3 time points (Supplemental Table 1).

The ability of Sytox Green to enter cells, bind DNA, and consequently fluoresce was used as another assay to assess membrane integrity [2, 3]. Cultures (5 mL) were grown overnight (37°C, 16-18 h) or to an OD_{600} for log phase cultures, 1mL was centrifuged (Microcentrifuge, 16 000 *g*),

resuspended in 600 μL 10 mM Tris-HCl, pH 8.0, and 3 μM Sytox Green (Invitrogen) dissolved in DMSO was added. The mixture was incubated in the dark for 10 min at room temperature and fluorescence was measured (excitation: 500 nm, emission: 550 nm). The fluorescence values were divided by the OD_{600} of the original culture and this value was divided by the fluorescence of the WT strain to determine relative fold fluorescence change (Supplemental Table 1).

A ToxiLight bioassay kit (Lonza) was used to assess membrane integrity by detecting the amount of adenylate kinase in the culture supernatant [2, 4]. Cultures (5 mL) were grown overnight (37°C, ~16 h) or to an OD_{600} for log phase cultures. All samples were diluted 10-fold with LB, and 100 μl was placed in a white 96 well plate (in duplicate). To prepare heat killed cells for a positive control, a 5 mL culture was grown overnight (stationary phase) or to an OD_{600} of ~0.4 (log phase), pelleted in a microfuge (10 000 *g*, 5 min, room temperature), resuspended in 1 mL sterile deionized water, and boiled for 3 min followed by sterile filtering (0.45 μm Ultra-free spin column filters, Millipore); lysates were diluted 100-fold and 100 μl was placed in a white 96 well plate (in duplicate). To all sample wells, 100 μl of ToxiLight reagent was added, and the mixture incubated at room temperature for 30 min. Luminescence was measured with a Molecular Devices SpectraMAX GeminiXS spectrometer. The average value of the duplicate sample Luminescence Units (LU) was multiplied by the dilution

factor and divided by the OD_{600} value of the original culture to account for differences in culture density (LU/OD). The adenylate kinase concentration was considered below detection (BD) when the measurement was negative (Supplemental Table 1).

Supplemental Table 1: Growth and membrane integrity phenotypes of strains.

Strains/ Treatments[1]	OMV Production[2]	Growth[3]	Growth w/ Actinomycin D[4]	Sytox Green[5]	Adenylate Kinase[6]
Log phase cultures:					
ΔycfSΔybiSΔerfK	1	WT	WT	WT	+
ΔycfSΔybiSΔerfKΔnlpA	3	WT	WT	<WT	+
ΔompA	1	WT	WT	WT	+
ΔnlpAΔompA	2	WT	WT	WT	BD
Stationary phase/overnight cultures:					
WT	1	WT	WT	WT	++
ΔycfSΔybiSΔerfK	44	WT	WT	WT	+
ΔycfSΔybiSΔerfKΔnlpA	63	WT	WT	WT	+
ΔompA	26	WT	WT	>WT	+
ΔnlpAΔompA	33	WT	WT	>WT	+
ΔampGΔamiD	14	WT	WT	WT	++
ΔampGΔnlpAΔamiD	11	WT	WT	WT	++
ΔmepAΔdacBΔpbpG	3	WT	WT	WT	++
ΔynhGΔycbB	0.4	WT	WT	<WT	+
ΔrfaC	10	<WT	WT	WT	BD
ΔrfaG	13	<WT	WT	WT	BD
ΔrfaP	15	<WT	WT	WT	BD

[1]Sets of assays performed in the same experimental group are separated by a space in the table; heat-killed cells were used as a positive control for Sytox Green and adenylate kinase activity; each assay was repeated at least twice.

[2]Fold-change in OMV production relative to WT or untreated. See Figures for statistical evaluations.

[3]Growth in LB: WT, like WT; <WT, below WT.

[4]Growth in LB with 5 μg/mL Actinomycin D: WT, like WT.

[5]Sytox Green entry: WT, no significant difference from WT; <WT, significantly lower compared to WT; >WT, significantly higher compared to WT.

[6]Adenylate kinase in supernatant:+, ~100-999 LU/OD; ++, ~1000-9999 LU/OD; For comparison, heat killed cells yielded >40,000 LU/OD for log phase cells and >20,000 LU/OD for stationary phase; BD, below detection, refers to samples with a negative value.

References:

1. Leive L: **Actinomycin Sensitivity in Escherichia Coli Produced by EDTA**. *Biochem Biophys Res Commun* 1965, **18**:13-17.

2. Schwechheimer C, Kuehn MJ: **Synthetic Effect between Envelope Stress and Lack of Outer Membrane Vesicle Production in Escherichia coli**. *J Bacteriol* 2013, **195**(18):4161-4173.

3. Cowles CE, Li Y, Semmelhack MF, Cristea IM, Silhavy TJ: **The free and bound forms of Lpp occupy distinct subcellular locations in Escherichia coli**. *Mol Microbiol* 2011, **79**(5):1168-1181.

4. Jacobs AC, Didone L, Jobson J, Sofia MK, Krysan D, Dunman PM: **Adenylate kinase release as a high-throughput-screening-compatible reporter of bacterial lysis for identification of antibacterial agents**. *Antimicrob Agents Chemother* 2013, **57**(1):26-36.

Supplemental Figure 1

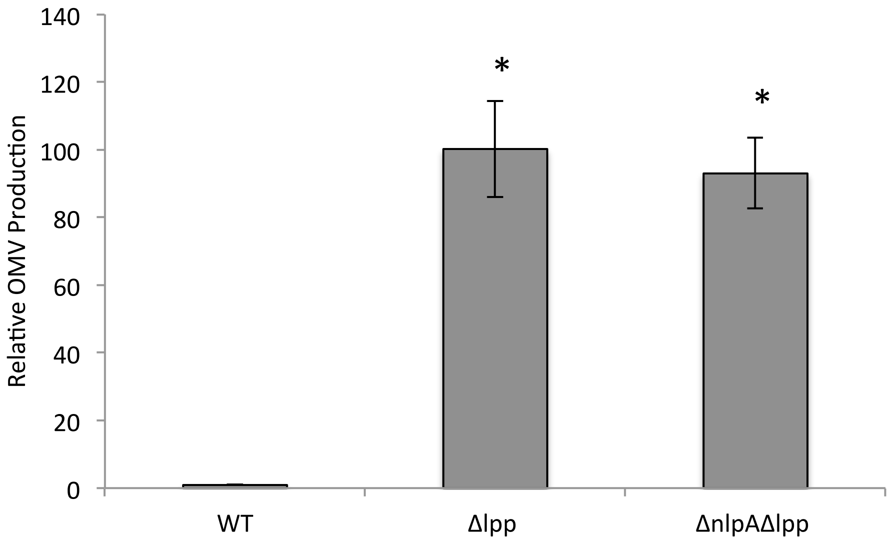

Supplemental Figure 1 Δ*lpp* hypervesiculation is not *nlpA* dependent
Relative fold OMV production in cultures of the indicated strains grown in LB overnight at 37°C was determined by quantitating OMVs, normalizing to OD_{600}, and dividing by OD_{600}-normalized OMV production in a WT culture. Error bars indicate standard error of the mean (SEM). *,$p \leq 0.05$; $n=2$

Supplemental Figure 2

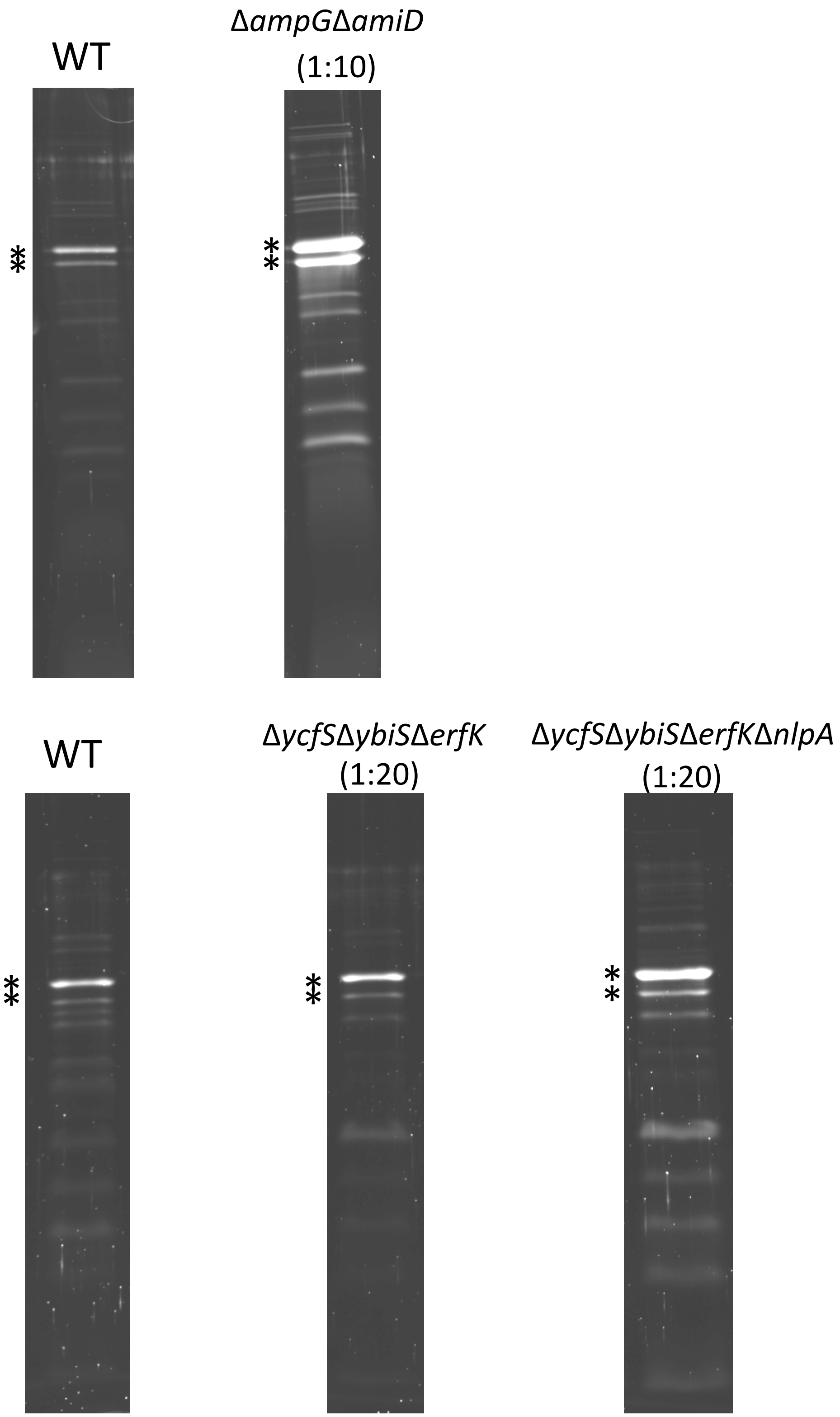

Supplemental Figure 2 Representative samples used for OMV quantitation Purified OMVs (from indicated strains) were diluted (if indicated), separated using 15% SDS-PAGE, and Ruby stained. Bands with asterisks (OmpsF/C/A) were quantitated.
